# Quasi-In-Situ Analysis of As-Rolled Microstructure of Magnesium Alloys during Annealing and Subsequent Plastic Deformation

**DOI:** 10.3390/ma15196581

**Published:** 2022-09-22

**Authors:** Jiafei Deng, Jing Tian, Yancai Zhou, Yuanying Chang, Wei Liang, Jinyao Ma

**Affiliations:** 1 College of Materials Science and Engineering, Taiyuan University of Technology, Taiyuan 030024, China; 2 Shanxi Key Laboratory of Advanced Magnesium-Based Materials, Taiyuan University of Technology, Taiyuan 030024, China; 3 Instrumental Analysis Center, Taiyuan University of Technology, Taiyuan 030024, China

**Keywords:** plastic behavior, recrystallization, rolled structure, ledges, cracks

## Abstract

In this paper, quasi-in situ experiments were carried out on rolled AZ31 magnesium alloy sheets to track the recrystallization behavior of the rolled microstructure during the heat treatment process and the plastic deformation behavior during the stretching process. The as-rolled microstructures are classified into five characteristics and their plastic deformation behaviors are described. The research shows that annealing recrystallization leads to grain reorganization, resulting in the diversity of grain orientation, and it is easier to activate basal slip. Recrystallization preferentially nucleates in the regions with high stress, while it is difficult for recrystallization to occur in regions with low stress, which leads to the uneven distribution of the as-rolled structure of magnesium alloys. Slip can be better transmitted between small grains, while deformation between large and small grains is difficult to transmit, which can easily lead to the generation of ledges. Incomplete recrystallization is more likely to accumulate dislocations than complete recrystallization, and ledges are formed in the early stage of deformation. Microcracks are more likely to occur between strain-incompatible grains. It is of great significance to promote the application of rolled AZ31 magnesium alloys for the development of heat treatment and subsequent plastic working of rolled magnesium alloys.

## 1. Introduction

Magnesium and magnesium alloys have a series of excellent properties such as low density, high specific strength, high specific height, good electrical conductivity and thermal conductivity, strong electromagnetic shielding ability and shock absorption ability, good processing performance, easy recycling, and are considered to be the most promising green metal materials in the 21st century [1,2,3,4,5]. For a long time, magnesium and magnesium alloys have been widely used in the field of aerospace, avionics, and automobile roofs, which can effectively reduce the weight of structural parts, save energy and reduce environmental pollution [6,7,8,9,10].

Magnesium alloy sheets are usually produced by rolling [11,12], and various forms of products can also be prepared by secondary forming methods such as stamping, showing extremely broad application prospects. The rolling process can refine the grains, improve the structure of magnesium alloys, and significantly improve the mechanical properties of magnesium alloys [13]. However, due to the close-packed hexagonal structure of magnesium, and the strong (0002) basal texture is easily formed during the rolling process of the sheet [14,15], few slip systems can be activated under common conditions [16], and rolling will lead to defects in the sheet [17]. When conventional rolling is used to produce magnesium alloy sheets, there are problems such as a long rolling process, low yield, and high production cost [18], and the sheet after rolling has high hardness and insufficient ductility. Therefore, annealing heat treatment is a necessary process for the rolled magnesium alloy sheet.

The recrystallization behavior of magnesium alloys has been extensively discussed by researchers [17,19,20,21,22,23,24]. The crystallographic defects generated in the rolling process of magnesium alloys can be disappeared through recrystallization annealing, and the grains can be re-formed to eliminate the deformation strengthening effect and residual stress, improve the plasticity, and restore the plastic deformation ability to facilitate further deformation processing [25]. Guan et al. [26,27,28] discussed the recrystallization behavior at the twinning and shear bands of cold-rolled WE43 magnesium alloys, respectively. Moreover, the study showed that the shear bands are preferential nucleation sites, and the shear band recrystallization is the main source of the observed recrystallized texture. The recrystallization of deformation twins accounts for a negligible proportion of the final recrystallized region. This is due to the low density of deformed twins and the intersections between these deformed twins. Our previous research also found that annealing can improve the uniformity of the deformed structure of wrought magnesium alloys by affecting the sequence of recrystallization in dense areas of the deformed structure and grain growth in sparse areas of the deformed structure [7].

In this paper, the quasi-in situ heating EBSD and quasi-in situ tensile EBSD tests are used to track the recrystallization behavior of as-rolled AZ31 magnesium alloy during the heat treatment process and the plastic deformation behavior during the stretching process. The as-rolled microstructure of magnesium alloy is divided into five categories according to the characteristics: (1) complete recrystallization and incomplete recrystallization, (2) the grain basal orientation and non-basal orientation, (3) large grain region and small grain region, (4) small and large strain regions and (5) potential crack-forming regions. The slip behaviors of five microstructures during plastic deformation are described, respectively. It is of great significance to promote the application of rolled AZ31 magnesium alloys for the development of heat treatment and subsequent plastic working of rolled magnesium alloys.

## 2. Materials and Methods

**Figure 1:**
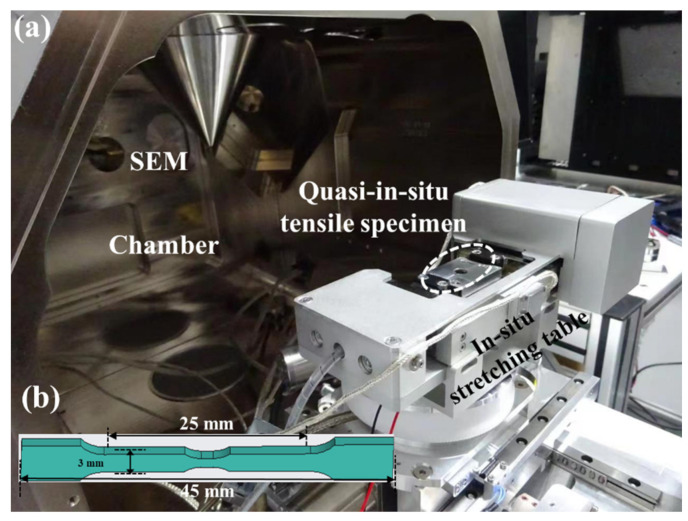
(**a**) In situ tension table and (**b**) in situ tensile specimen.

The starting material used in this work is hot-rolled AZ31 magnesium alloy sheet. We used an EDM to prepare a block specimen with dimensions of 4 (rolling direction, RD) × 8 mm (transverse direction, TD) × 3 mm (normal direction, ND) and a tensile specimen of 35 mm (RD) × 6 mm (TD) × 1 mm (ND), the morphology of the tensile specimen is shown in Figure 1b.

### 2.1. Quasi-In Situ Heating EBSD Test

The RD-ND planes of the block specimen were observed using a field emission scanning electron microscope (SEM; S8000G, TESCAN, Brno, Czech Republic) equipped with EDS and electron backscatter diffraction (EBSD) systems. The bulk sample was used for quasi-in-situ heating EBSD analysis, that is, the same position of the sample was observed for EBSD after heating for 0-min, 10 min, and 30 min at 200 °C. The EBSD observation area was 320 μm × 240 μm, the step size was set to 0.9 μm. The voltage was set to 20 keV and the current was 3 nA.

### 2.2. Quasi-In Situ Tensile EBSD Test

Tensile specimens were used for quasi-in situ tensile EBSD analysis, that is, SEM and EBSD observations were performed on the same position of the specimen at different strain stages (Original stage: S0, yield stage: S1, fracture stage: S2) when stretched along the RD. The observation surface is the RD-TD plane, the observation area is 230 μm × 180 μm, and the step size is set to 0.25 μm. Figure 1a shows the in situ tension table. In situ tensile tests were performed at room temperature with a constant displacement rate of 0.001 mm/s (approximate strain rate of 2.5 × 10^−4^ s^−1^) using a screw-driven tensile stage placed in an S8000G chamber. To ensure the clamping style of the tension table, a preload 20 N was applied before loading.

The samples used for EBSD analysis were sequentially ground with 25 μm, 10 μm, 5 μm, 3 μm, 1.5 μm, and 0.5 μm grit sandpaper until the surface of the samples was smooth. The ground samples are electropolished with 5% perchloric acid and 95% alcohol solution. The electrolysis temperature is between −20 °C and −40°C, the electrolysis time is 1 min, the electrolysis voltage is 45 V, and the current is about 0.35 A. The surface of the sample after electropolishing is bright and without deep pits. Finally, Channel 5 software was used to process the obtained EBSD data and subsequent slip trace analysis.

Based on SEM images and EBSD data acquired during tensile testing, slip trace analysis was performed to identify activated slip systems. The following common slip systems in magnesium alloys are considered [18,29,30,31,32]:

(1) $\left\{0001\right\}\langle 11\bar{2}0\rangle$: basal<a>;
(2) $\left\{10\bar{1}0\right\}\langle 11\bar{2}0\rangle$: prismatic<a>;
(3) $\left\{01\bar{1}1\right\}\langle 11\bar{2}0\rangle$: first-order pyramidal<a>;
(4) $\left\{11\bar{2}2\right\}\langle 11\bar{2}\bar{3}\rangle$: second-order pyramidal<c+a>.

Combining the slip traces observed inside specific grains in the SEM images, and the average grain orientation obtained from the corresponding EBSD, all possible slip surface traces and the Schmid factor values of the corresponding slip systems were calculated using the self-made MATLAB code. Based on the best match between the observed slip traces and the calculated results, a specific slip system is selected as the active slip system. Furthermore, some identical slip modes share the same slip plane, such as all basal <a> slip systems, and slip trace analysis cannot distinguish them. In this case, the slipping system that exhibits the largest Schmid factor value is considered the active system.

## 3. Results and Analysis

### 3.1. Recrystallization Behavior of the As-Rolled Microstructure

**Figure 2:**
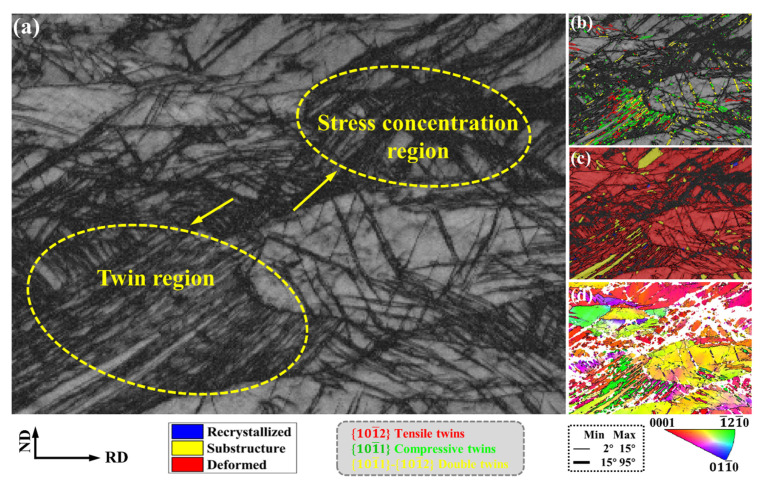
The EBSD result of the as-rolled structure of magnesium alloy: (**a**) BC map; (**b**) distribution of different twins; (**c**) distribution of different microstructures; and (**d**) IPF map.

Figure 2 is the EBSD result of the as-rolled structure of AZ31 magnesium alloy, Figure 2a is the BC (band contrast) map, Figure 2b is the distribution of different twins, Figure 2c is the deformed structure, recrystallization and substructure distribution, and Figure 2d is the IPF map, showing the orientation of the as-rolled microstructure of the magnesium alloy. After the magnesium alloy is rolled, the deformed structure increases and almost no recrystallization occurs. As a typical as-rolled structure of magnesium alloys, twinning, and shear bands are abundantly generated, as shown in Figure 2a,b. Compressive twins and double twins are more likely to be generated during the rolling process of magnesium alloys and have slender morphologies. At the same time, some deformed structures cannot be analyzed by EBSD due to stress concentration.

**Figure 3:**
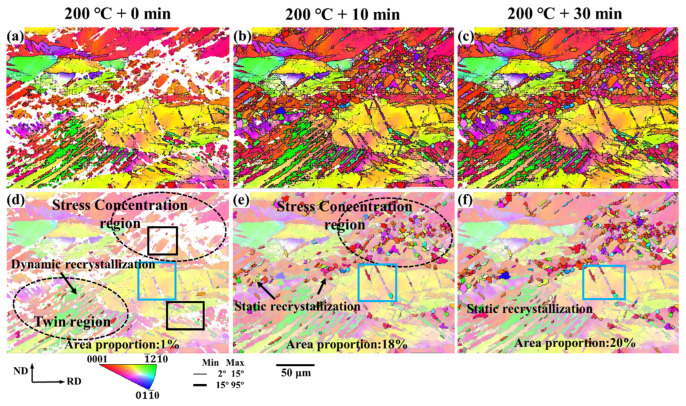
The microstructure evolution of the as-rolled magnesium alloy during in-situ heating: (**a**–**c**) IPF maps after different annealing conditions; (**d**–**f**) Recrystallization distribution after different annealing conditions. The resulting static recrystallization is highlighted.

Figure 3 shows the microstructure evolution of the as-rolled AZ31 magnesium alloy during in situ heating. Figure 3a–c are the microstructures of unannealed, annealed at 200 °C for 10 min, and annealed at 200 °C for 30 min, respectively. Figure 3d–f show the distribution of recrystallization under different annealing conditions, respectively, and the resulting static recrystallization is highlighted.

In the as-rolled structure without heat treatment, only a small amount of dynamic recrystallization occurs in the twin region. After annealing at 200 °C for 10 min, the proportion of recrystallized area gradually increased from 1% to 18% and after 30 min of annealing, the proportion of recrystallized area was further increased to 20%.

More static recrystallization occurs in the stress concentration region (as shown by the black dotted circle in Figure 3d,e), and the number of static recrystallizations increases gradually with the increase in annealing time. There is almost no static recrystallization behavior in the twinned region, while part of the twin tips appears in the recrystallization behavior, as shown in the blue solid line box in Figure 3d–f.

After heat treatment of rolled magnesium alloy sheets, annealing recrystallization leads to grain reorganization, and recrystallization preferentially nucleates in areas with high stress, and the generation of fine and equiaxed recrystallization contributes to grain refinement. However, for regions with small stress and large grains, recrystallization is difficult to occur, and some grains have incomplete recrystallization. This results in uneven distribution of the as-rolled microstructure of magnesium alloys. At the same time, annealing and recrystallization lead to grain reorganization, and the grain orientation is more diverse.

**Figure 4:**
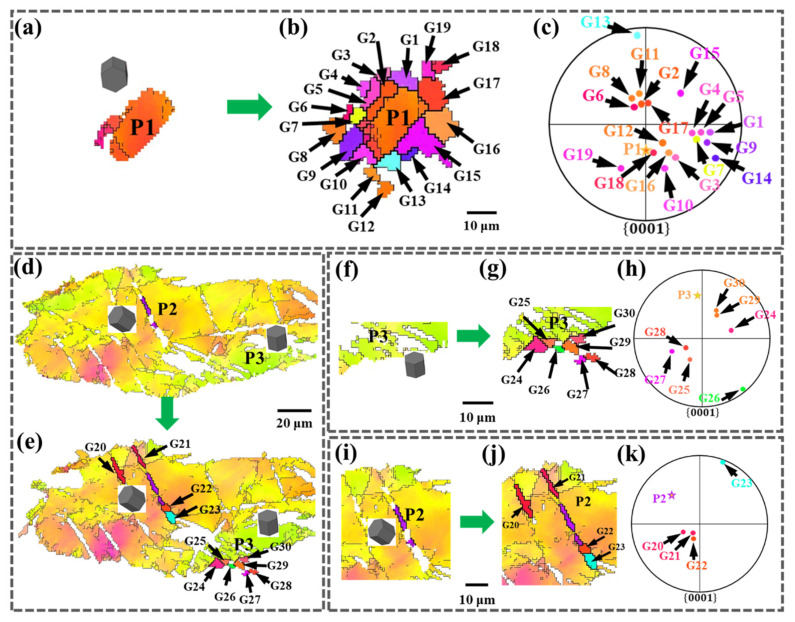
Microstructure evolution of different regions of as-rolled magnesium alloy during quasi-in situ heating: (**a**,**d**,**f**,**i**) 0 min, (**b**,**e**,**g**,**j**) 30 min, (**c**,**h**,**k**) {0001} pole figure.

To analyze the static recrystallization behavior of the stress concentration region, the region in the black solid line box in Figure 3d is enlarged, as shown in Figure 4a,f. Figure 4a,d,f, are the as-rolled microstructures of magnesium alloys before annealing and Figure 4b,e,g are the recrystallized morphologies of the as-rolled structure after annealing at 200 °C for 30 min. The distributions of recrystallized grains in the {0001} pole figure are shown in Figure 4c,h, respectively.

After annealing, the parent grains P1 have similar orientations to a small amount of static recrystallized grains (G2, G8, G11, G12, G16), but are quite different from other recrystallized grains. Meanwhile, the {0001} pole figure shown in Figure 4c shows that only a few grains are distributed around P1, and most of the recrystallized grains are randomly distributed in the {0001} pole figure and deviate from the parent grains. The parent grains P3 are oriented differently from the static recrystallized (G24–G30), and the {0001} pole figure shown in Figure 4h shows that all the recrystallized grains are not around P3. The above results indicate the diversity of recrystallization orientations in the stress concentration region.

The static recrystallization behavior of the twin tip region is further analyzed, and the region in the solid blue box in Figure 3d,f are enlarged in Figure 4i,j, and the corresponding IPF maps are shown. The distribution of recrystallized grains in the {0001} pole figure is shown in Figure 4k. After annealing, the tip of twinned P2 produces static recrystallization (G21 and G22) with a large difference in orientation, while the orientations between recrystallizations are similar. From the {0001} pole figure, the c-axis of the recrystallized grains is approximately parallel to the ND, and the crystal orientation is the basal plane orientation.

According to the quasi-in situ heating analysis, the rolled microstructure of AZ31 magnesium alloy after heat treatment has the following five characteristics: (1) Due to the uneven structure of the rolled magnesium alloy, at the same annealing temperature, recrystallization occurs preferentially in the large stress concentration area, and then the grain grows, while the energy in the small stress concentration area may not be enough for recrystallization. Therefore, as the annealing time increases, there is always incomplete recrystallization. (2) Recrystallization leads to changes in grain orientation, and most of the as-rolled structures are basal plane orientation, while recrystallization will cause the orientation to deviate from the parent grain, resulting in the diversity of grain orientation. (3) Due to the uneven distribution of the structure, there are large grain regions and small grain regions. (4) The strain distribution of the as-rolled structure is uneven, and there are large strain areas and small strain areas. (5) Potential crack-forming regions during subsequent plastic deformation.

### 3.2. Plastic Deformation Behavior of Rolled Microstructure

**Figure 5:**
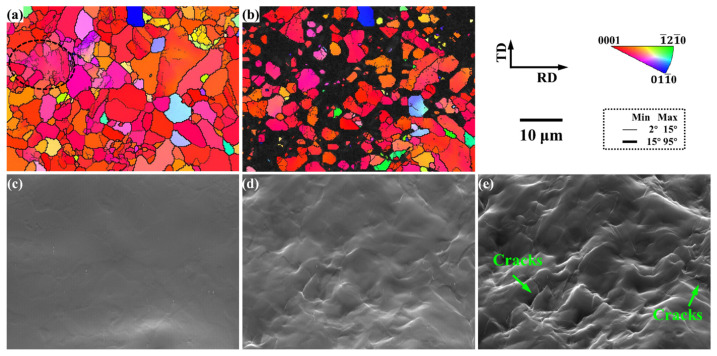
Microstructure evolution of the quasi-in situ tensile test of the as-rolled microstructure of magnesium alloys at different strain stages: (**a**,**c**) Original stage: S0, (**b**,**d**) yield stage: S1, (**e**) fracture stage: S2.

To further explore the plastic deformation behavior of the as-rolled AZ31 microstructure during the tensile process, quasi-in situ tensile EBSD experiments were carried out. Figure 5 shows the microstructure evolution of the quasi-in situ tensile test of the as-rolled microstructure of magnesium alloys. The results show that the grain orientation is unchanged, and cracks are generated along the grain boundaries. The as-rolled microstructure of magnesium alloy is divided into five parts: complete recrystallization and incomplete recrystallization, basal orientation grain and non-basal orientation grain, large grain and small grain, small strain area and large strain region, and crack-forming region and their plastic deformation behavior during stretching is explored, respectively.

#### 3.2.1. Complete Recrystallization and Incomplete Recrystallization

**Figure 6:**
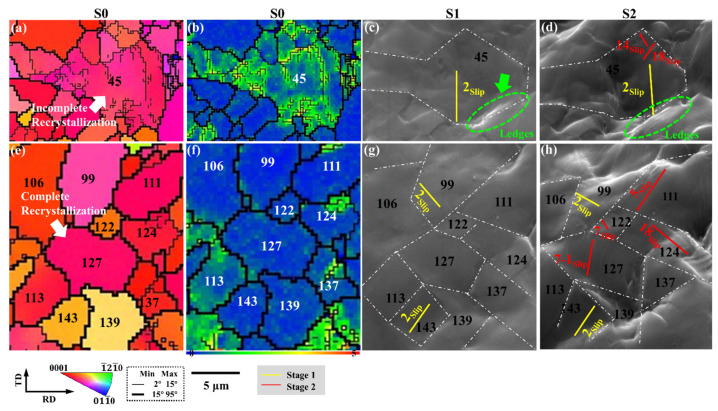
The plastic behavior of complete recrystallization and incomplete recrystallization during uniaxial stretching: (**a**,**e**) IPF images; (**b**,**f**) KAM images; and (**c**,**d**,**g**,**h**) SEM images at different strain stages. The slip system represented by the slip traces in the figure is shown in Figure 7.

Figure 6 shows the identification of the slip systems of complete recrystallization and incomplete recrystallization during uniaxial stretching, and shows the corresponding IPF images, KAM images, and SEM images at different strain stages.

The low-angle grain boundaries in the incomplete recrystallization G45 are densely distributed, and the KAM value is high. The basal slip system $\left(0001\right)\left[\bar{1}2\bar{1}0\right]$ is preferentially activated in the yield stage, and the strain is concentrated at the grain boundaries, and the ledge is formed, as shown by the green dotted circle in Figure 6c. As the load increases to the fracture stage, the lattice distortion in G45 increases, the ledges are further deepened, and the second-order pyramidal slip variants $\left(11\bar{2}2\right)\left[11\bar{2}\bar{3}\right]$ and $\left(1\bar{2}12\right)\left[1\bar{2}1\bar{3}\right]$ become active.

However, there are almost no low-angle grain boundaries in the fully recrystallized grains (G99, G127, G122, G139), and the KAM value is low. At the yield stage, the basal slip system is activated in G99. With the increase in strain, the non-basal slip system is activated in G127 and G122. At the same time, we found that the adjacent complete recrystallization (G99 and G122, G122 and G127, G127, and G139) was not easy to form ledges in the early deformation stage, and only a few ledges were formed in the later stage of deformation.

This is because there is less accumulation of original dislocations in the complete recrystallization, and it is not easy to accumulate dislocations in the early deformation stage, resulting in stress concentration. There are many original dislocations in incomplete recrystallization, and it is easier to accumulate dislocations, and ledges have been formed in the early stage of deformation.

#### 3.2.2. The Grain Basal Orientation and Non-Basal Orientation

**Figure 7:**
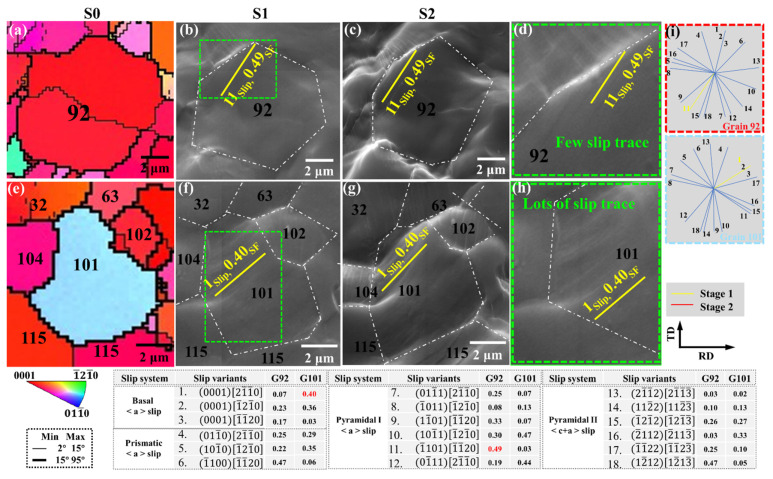
The plastic behavior of basal plane-oriented and non-basal plane-oriented grains during uniaxial stretching: (**a**,**e**) IPF images and (**b**–**d**,**f**–**h**) SEM images at different strain stages, (**i**) Slip traces for potential slip systems, with active slip systems highlighted.

Figure 7 is the identification of basal plane-oriented and non-basal plane-oriented grains in slip systems during uniaxial stretching, and shows the corresponding IPF images, as well as SEM images at different strain stages. The slip traces and corresponding Schmid factor values for all potential slip systems in G92 and G101 are visible in the figure, with the slip traces and corresponding Schmid factor for the activated slip system highlighted.

When the basal plane-oriented G92 is uniaxially stretched along the RD, a small amount of first-order pyramidal <a> slip $\left(\bar{1}101\right)\left[\bar{1}\bar{1}20\right]$ with the highest SF is preferentially activated, and its value is 0.49. In the later stages of deformation, no new slip systems are activated. However, the non-basal-oriented grains G101 produced a large number of basal slip traces during the deformation process, which was due to the highest SF and low CRSS of the basal slip, and the basal slip was easily activated. In the later stretching process, no new slip traces were generated, and the slip traces of the basal slip increased, indicating that the basal slip was more fully activated.

When the rolled structure is annealed, the recrystallization causes the diversity of grain orientation, and some grains deviate from the orientation of the basal-oriented parent grain, showing a non-basal orientation. At room temperature, the CRSS of basal slip is low and easy to activate [33], and in the grains with non-basal orientation, the Schmid factor of activating basal slip is generally high.

Therefore, there are lots of basal slip traces in the non-basal oriented grains, and the basal slip is fully activated. According to Schmid’s law [34], the pyramidal slip should be easier to start in grains with basal orientation. However, there are only a few pyramidal slip traces in basal-oriented grains, and even in the later stage of deformation, the slip traces do not increase, which should be limited by the high CRSS of pyramidal slip.

#### 3.2.3. Large Grain Region and Small Grain Region

**Figure 8:**
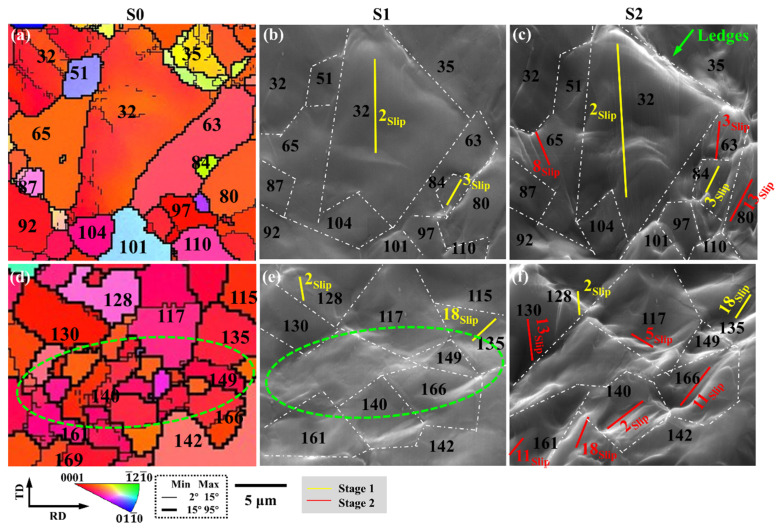
The plastic behavior of large-grained and small-grained regions in slip systems during uniaxial stretching: (**a**,**d**) IPF images and (**b**,**c**,**e**,**f**) SEM images at different strain stages. The slip system represented by the slip traces in the figure is shown in Figure 7.

Figure 8 demonstrates the identification of the slip systems in the large-grained and small-grained regions during uniaxial stretching, and shows the corresponding IPF images, as well as SEM images at different strain stages.

In the large grain region, G32 activates the basal slip at the initial stage of deformation, and as the strain increases to stage 2, G63, G65, and G80 activate the prismatic and pyramidal slip to coordinate the deformation. In the small grain area, basal slip and second-order pyramidal slip are preferentially activated in G128 and G135, and then basal slip, prismatic, and pyramidal slip are activated in stage 2 to coordinate the subsequent plastic deformation.

Grain size has little effect on the type of activated slip system. However, the small-grain region activates more slip systems than the large-grain region, as shown by the green dashed box in Figure 8e. Due to the small grain size, the slip system can be better transferred between grains. However, in the large-grain region, it is difficult to transfer the deformation between the large and small grains, which can easily lead to the generation of ledges.

#### 3.2.4. Small and Large Strain Regions

**Figure 9:**
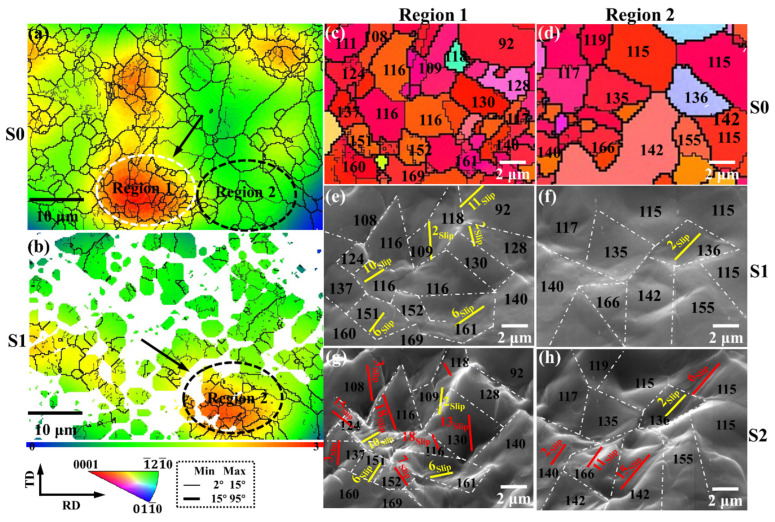
The plastic behavior of the small and large strain regions during uniaxial stretching: strain distribution of (**a**) S0 and (**b**) S1, (**c**,**d**) IPF images, and (**e**–**h**) SEM images at different strain stages. The slip system represented by the slip traces in the figure is shown in Figure 7.

Figure 9 demonstrates the identification of the slip systems in the small and large strain regions during uniaxial stretching, and shows the corresponding strain distribution maps, IPF maps, and SEM images at different strain stages.

Figure 9a is the strain distribution of the microstructure before uniaxial stretching. Before deformation, the large strain region is concentrated in region 1 with small grains, while the strain distribution in region 2 with larger grains is less. As the deformation increases (Figure 9b), region 2 gradually transforms into a large-strain region, while the original high-strain region 1 becomes higher due to the strain after stretching, and the lattice distortion becomes more serious so that the EBSD resolution of its corresponding region decreases.

At stage 1, region 1 with smaller grains activated more grains of the slip system, while region 2 with larger grains activated a smaller number of slip systems. When the deformation reaches stage 2, the activated slip systems in both regions 1 and 2 increase, and the small-strain region transforms into a large-strain region.

#### 3.2.5. Crack-Forming Region

**Figure 10:**
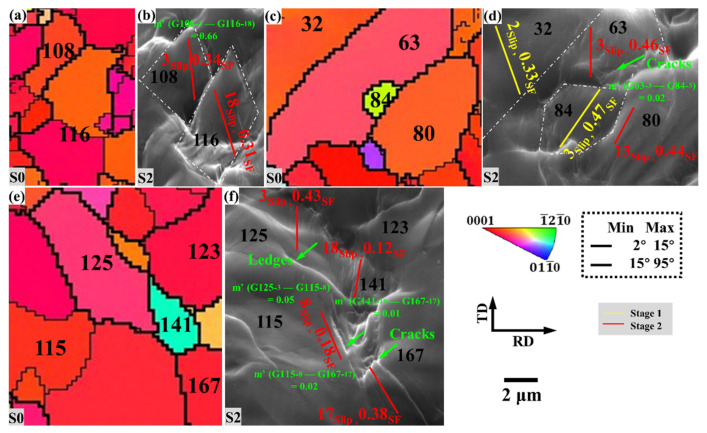
The slip traces of the specimen near the microcrack during uniaxial tension.: (**a**,**c**,**e**) IPF images at S0, and (**b**,**d**,**f**) SEM images at S2. The slip system represented by the slip traces in the figure is shown in Figure 7.

Figure 10 shows the slip traces of the specimen near the microcrack during uniaxial tension, and the plastic deformation behavior of the grains in the three regions is analyzed. Meanwhile, to analyze the degree of strain compatibility between different slip systems in the slip transfer between adjacent grains in the region, the Lustre–Morris parameter (geometric compatibility factor) is defined as follows [35,36]:

m′=cosκcosψ

The angles κ and ψ, respectively, denote the angle between the slip directions of the two slip systems involved and that between the normal directions of the two slip planes.

There is an intergranular fracture between grains G108 and G116, and the Schmid factor values of G108-activated basal slip and G116-activated second-order pyramidal slip are higher, and the geometric compatibility between them is relatively high, and *m*’ is 0.66, but it’s still not enough to coordinate the strain here.

The activated slip systems of grains G32, G63, G84, and G80 are all high Schmid factor values, indicating that the local strain here is consistent with the macroscopic strain. The generation of microcracks between G63 and G80 is due to the extremely low geometric compatibility factor. Even if the Schmid factor value is high, the slip system is easy to activate, but the incompatibility of strain between adjacent grains can also lead to the concentration of stress, which develops into cracks. *m*’ between G125 and G115, G141 and G115, and G141 and G167 are 0.05, 0.02, and 0.01, respectively. More strain incompatibility between grain pairs will lead to the concentrated generation of numerous micro-cracks, which expand into the crack.

## 4. Conclusions

1. Annealing recrystallization leads to grain reorganization, resulting in the diversity of grain orientation. Recrystallization preferentially nucleates in regions with high stress, and the generation of fine and equiaxed recrystallization contributes to grain refinement. However, it is difficult for recrystallization to occur in the region with less stress, which leads to the uneven distribution of the as-rolled structure of AZ31 magnesium alloys.
2. The microstructure transformation and cracking of as-rolled AZ31 magnesium alloy during isothermal annealing and tensile deformation have the following characteristics:
  (1) Incomplete recrystallization is easier to accumulate dislocations than complete recrystallization, and ledges are formed in the early stage of deformation
  (2) The grain with non-basal orientation is easier to activate basal slip than basal-oriented grains.
  (3) Slip can be better transmitted between small grains, while deformation between large and small grains is difficult to transmit, which can easily lead to the generation of ledges.
  (4) The initial large-strain region is concentrated near the small-sized grains, and slip is preferentially activated in the early deformation stage, and the large-strain region transforms in the later stage of deformation.
  (5) Microcracks are more likely to occur between strain-incompatible grains.

## Data Availability

The data that support the findings of this study are available from the corresponding author upon reasonable request.
